# Crystal Structure and Substrate Specificity of _D_-Galactose-6-Phosphate Isomerase Complexed with Substrates

**DOI:** 10.1371/journal.pone.0072902

**Published:** 2013-08-28

**Authors:** Woo-Suk Jung, Raushan Kumar Singh, Jung-Kul Lee, Cheol-Ho Pan

**Affiliations:** 1 Functional Food Center, Korea Institute of Science and Technology Gangneung Institute, Gangneung, Korea; 2 Department of Chemical Engineering, Konkuk University, Gwangjin-Gu, Seoul, Korea; Griffith University, Australia

## Abstract

D-Galactose-6-phosphate isomerase from *Lactobacillus rhamnosus* (LacAB; EC 5.3.1.26), which is encoded by the tagatose-6-phosphate pathway gene cluster (*lacABCD*), catalyzes the isomerization of D-galactose-6-phosphate to D-tagatose-6-phosphate during lactose catabolism and is used to produce rare sugars as low-calorie natural sweeteners. The crystal structures of LacAB and its complex with D-tagatose-6-phosphate revealed that LacAB is a homotetramer of LacA and LacB subunits, with a structure similar to that of ribose-5-phosphate isomerase (Rpi). Structurally, LacAB belongs to the RpiB/LacAB superfamily, having a Rossmann-like αβα sandwich fold as has been identified in pentose phosphate isomerase and hexose phosphate isomerase. In contrast to other family members, the LacB subunit also has a unique α7 helix in its C-terminus. One active site is distinctly located at the interface between LacA and LacB, whereas two active sites are present in RpiB. In the structure of the product complex, the phosphate group of D-tagatose-6-phosphate is bound to three arginine residues, including Arg-39, producing a different substrate orientation than that in RpiB, where the substrate binds at Asp-43. Due to the proximity of the Arg-134 residue and backbone Cα of the α6 helix in LacA to the last Asp-172 residue of LacB with a hydrogen bond, a six-carbon sugar-phosphate can bind in the larger pocket of LacAB, compared with RpiB. His-96 in the active site is important for ring opening and substrate orientation, and Cys-65 is essential for the isomerization activity of the enzyme. Two rare sugar substrates, D-psicose and D-ribulose, show optimal binding in the LacAB-substrate complex. These findings were supported by the results of LacA activity assays.

## Introduction

Lactose, which is found mainly in cow’s milk, is a disaccharide derived from β-D-galactose and α/β-D-glucose, and is fermented via multiple pathways in Gram-positive bacteria such as *Streptococci* [1], *Staphylococci* [2–4], and *Lactococci* [5]. In most Gram-positive bacteria, lactose is initially transported and phosphorylated to lactose-6-phosphate (Lac6P) by the phosphoenolpyruvate (PEP)-dependent sugar phosphotransferase system (PTS) [2]. Some bacteria such as *Escherichia coli* and *Lactococcus lactis* can transfer lactose through a non-PTS transporter [6,7]. In bacteria that utilize lactose as a carbohydrate source, the disaccharide is either converted to glucose through the Leloir pathway [8] and then metabolized by glycolysis [1], or catabolized directly through the D-tagatose-6-phosphate pathway [5]. In lactic acid bacteria, β-galactosidase hydrolyzes Lac6P to form glucose and D-galactose-6-phosphate (Gal6P) [6,7]. Gal6P is then catabolized by the sequential enzymatic activity of galactose-6-phosphate isomerase (LacAB), tagatose-6-phosphate (Tag6P) kinase (LacC), and tagatose-1,6-diphosphate aldolase (LacD), which are all encoded by the tagatose-6-phosphate pathway gene cluster (*lacABCD*) [5,9–12]. Dihydroxyacetone phosphate and D-glyceraldehyde-3-phosphate are the final products of Gal6P catabolism via the tagatose-6-phosphate pathway [4,13].

**Figure 1:**
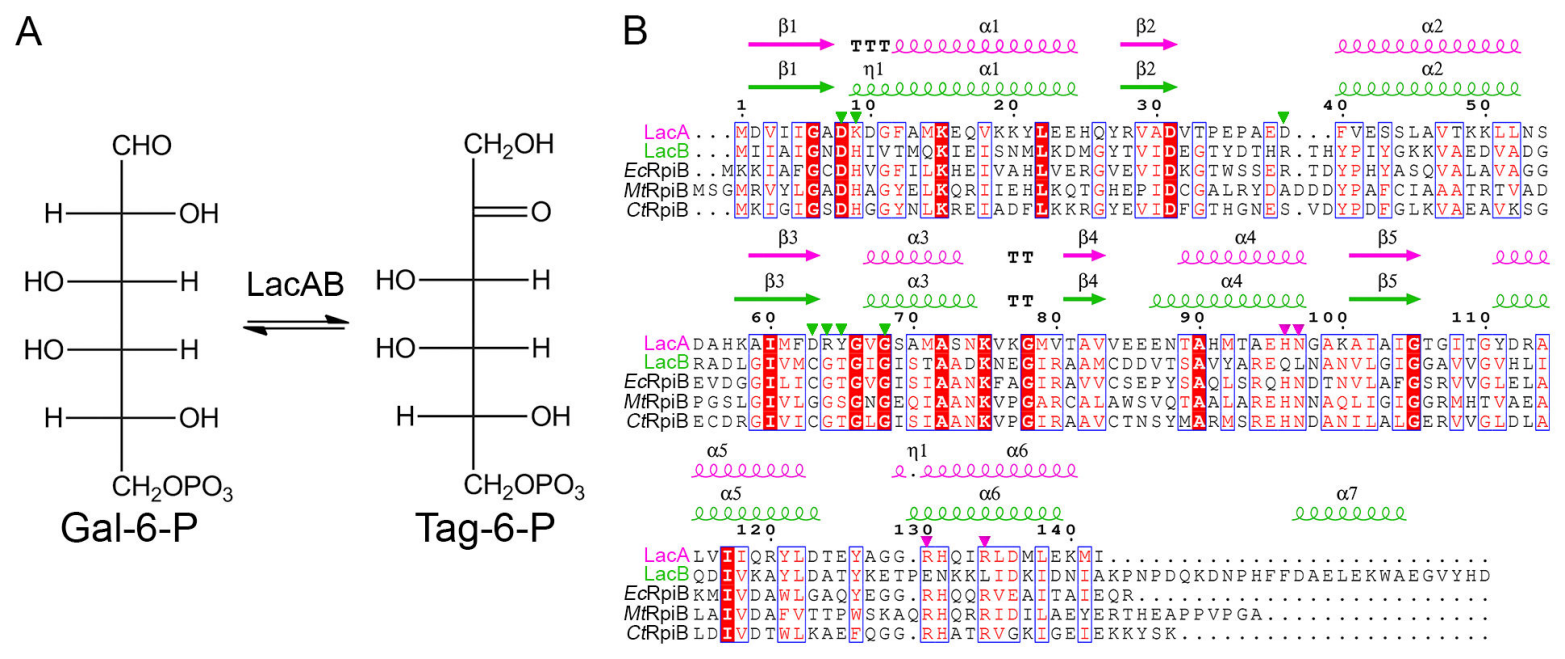
Schematic of the reaction catalyzed by LacAB and alignment of LacA, LacB, and RpiB sequences. **A**, Galactose-6-phosphate (Gal6P) is converted to tagatose-6-phosphate (Tag6P) by LacAB during lactose catabolism. **B**, Multiple sequence alignment of LacA and LacB from *Lactobacillus rhamnosus* (GenBank accession numbers ZP03210387 and ZP03210388, respectively), *Ec*RpiB from *Escherichia coli* (PDB ID 1NN4; NP418514), *Mt*RpiB from *Mycobacterium tuberculosis* (2VVP; YP006515902), and *Ct*RpiB from *Clostridium thermocellum* (3PH4; YP001038990). The sequences are for precursors, and the numbering is based on LacA. Highly conserved residues are shown in red type and boxed in blue; strictly conserved residues are shown on a red background. Secondary structure elements are indicated in pink for LacA and in green for LacB. Residues interacting directly with bound Taga6P are indicated with a triangle in pink (LacA) and green (LacB). The figure was prepared using ESPript [41].

The three-dimensional structure and mechanism of action of tagatose-6-phosphate kinase (LacC) [14] and of class I and II tagatose-1,6-diphosphate aldolase (LacD) [15,16] have been investigated using X-ray crystallography techniques. Galactose-6-phosphate isomerase (LacAB), which is a heteromultimer of LacA and LacB subunits, has been cloned, expressed in E. coli, and shown to convert Gal6P to Tag6P (Figure 1A) [5,9,10]. LacA and LacB comprise 142 and 172 amino acids, respectively [5,9], and share 26% amino acid sequence identity with ribose-5-phosphate isomerase (RpiB; EC 5.3.1.6), which converts ribose-5-phosphate to ribulose-5-phosphate in the pentose phosphate pathway. Structurally, LacA and LacB belong to the RpiB/LacAB family, with a Rossmann-like αβα sandwich fold [17].

Several sugar phosphate isomerases, including ribose-5-phosphate isomerase [18,19], galactose-5-phosphate isomerase [20], mannose-6-phosphate isomerase [21], and glucose-6-phosphate isomerase [18], have been studied for their application in the production of rare sugars by the interconversion between aldoses and ketoses. Similar studies have examined the efficiency and substrate specificity of LacAB for the production of rare sugars such as D-psicose and D-ribulose from D-allose and D-ribose, respectively [20].

In the present study, to understand the structural features and substrate specificity of multimeric LacAB, we determined the crystal structure of LacAB from *Lactobacillus rhamnosus* in its native form and in a complex with tagatose-6-phosphate as a product or with D-allose and D-ribose as substrates. We analyzed the active site residues using site-directed mutagenesis and simple enzyme activity assays.

## Results

### Overall structure of the LacAB

For co-expression of the LacA and LacB genes, each gene was obtained from the lactose operon and subcloned into pQE-80L vector containing two Shine-Dalgarno sequences as ribosomal binding sites. For structural and kinetic studies, LacA and LacB proteins were overexpressed as His-tagged proteins in E. coli. The purified LacA and LacB proteins gave bands of 15 kDa and 19 kDa, respectively, on sodium dodecyl sulfate-polyacrylamide gels.

Initially, the structure of LacAB was determined at a resolution of 1.96 Å using a crystal obtained from microseeding using the hanging drop vapor diffusion method. To obtain the LacAB-product complex, the crystals were soaked with tagatose-6-phosphate; X-ray data were collected at 1.65 Å resolution. The structure of the complex was resolved by molecular replacement with a refined substrate-free LacAB structure as the search model.

**Figure 2:**
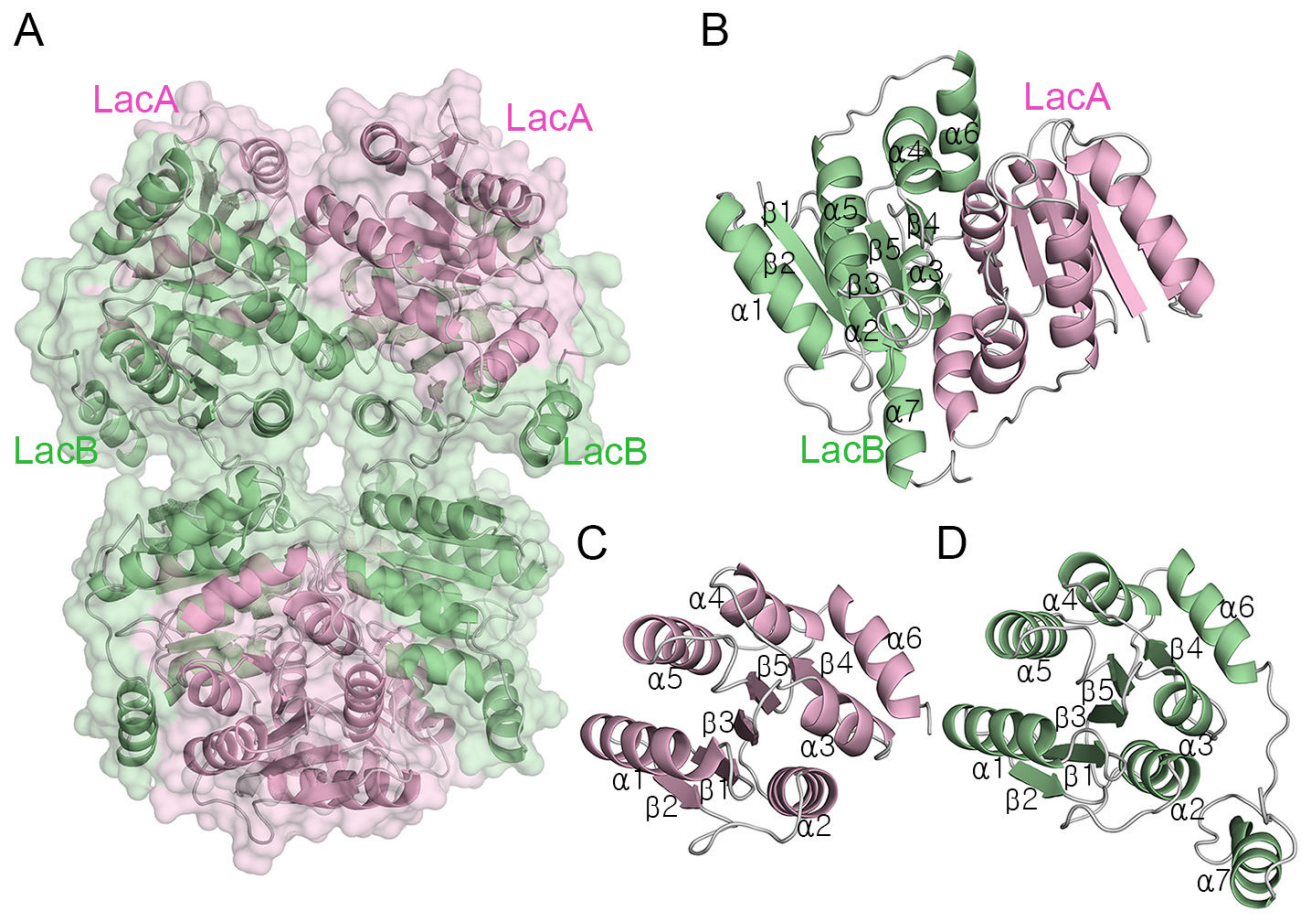
Overall structure of LacAB and each subunit. **A**, A ribbon diagram and transparent surface representation of LacAB as a homotetramer. **B**, A ribbon diagram of the LacAB monomer showing the secondary structure as defined in Figure 1B. **C**, A ribbon diagram of a LacA subunit showing the five parallel β-sheets in the center surrounded by five α-helices, with α1, α4, and α5 on the left, and α2 and α3 on the right. The α6 helix is located perpendicular to the α3β4α4 motif. **D**, A ribbon diagram of the LacB subunit, as described for LacA in Figure 2C. The extra C-terminal α7 helix is located nearly parallel to the α2 helix.

In the crystal structure, the two LacABs in asymmetric unit (subunit AB and CD) is related by non-crystallographic symmetry, and the whole LacAB homotetramer is arranged with crystallographic 2-fold symmetry of each two asymmetric units, which is shown in Figure 2A, has dimensions of 108 × 79 × 54 Å (Figure 2A and B). This interface is predicted by PISA [22], leading to stable homotetramer formation of LacAB in solution with a ΔG of dissociation of 7.8 kcal/mol. To confirm the solution state of LacAB, the molar mass was analyzed using an online size exclusion chromatography-fast protein liquid chromatography (SEC-FPLC) coupled with multi-angle light scattering (MALS) and UV detector. Figure S1 shows the UV280nm-LS overlay of LacAB. The column retention time in SEC was 13.62 min, and the MALS estimated molecular weight (Mw) of the major peak was about 135.5 kDa with a polydispersity (Pd) of 1.026.

Each subunit of LacA and LacB forms a Rossmann-like αβα sandwich fold, which was initially identified in pentose phosphate and hexose phosphate isomerases, including ribose-5-phosphate isomerases (RpiA and B), from many species [17,23,24]. As shown in Figure 2C and D, the structures of LacA and LacB subunits, like that of RpiB, contain five α-helices. Two α-helices (α2 and α3) are located on one side of a five-stranded parallel β-sheet with strands in the order β2, β1, β3, β5, and β4, and the other three (α1, α4, and α5) are on the other side. Another α-helix (α6) extends from the main αβα sandwich fold and is situated horizontally to the other helices. The LacB also contains a seventh α-helix (α7) in the C-terminus, which is 30 amino acids longer than the LacA C-terminus. A structure-based alignment of the αβα sandwich fold sequences, including ribose-5-phosphate isomerase sequences from various species, is presented in Figure 1B.

### Interface between LacA and LacB

The LacAB monomer involves face-to-face contacts between the respective flat interfaces of the two proteins and interactions between a helix of LacA and two helices of LacB located near the C-terminus. The LacAB interface has a total buried surface area of 2069 Å^2^ in LacA and 1895 Å^2^ in LacB, representing 21% of the total surface area of LacA and 28% of the total surface area of LacB, respectively. The third αβα folds (α3/β4/α4) of LacA and LacB are interlocked perpendicularly to each other (Figure S2). The two C-terminal helices of LacB are also perpendicular. The surfaces of the two proteins interact by direct polar contacts, 12 hydrogen bonds, and hydrophobic interactions among Val-67, Met-71, Val-80, Val-83, Thr-89, Met-92, and Thr-93 in LacA and Thr-67, Ile-69, Thr-73, Met-85, Thr-90, Leu-99, Val-110, and Ile-141 in LacB. In addition, the hydrophilic residues Asn-74, Glu-85, and Glu-86 in LacA and Ser-72, Asp-76, Asp-87, Asp-88, and Ser-91 in LacB are indirectly connected by seven intervening water molecules. The interactions between the C-terminal α-helix (α7) of LacB and the antiparallel α-helix (α6) of LacA create a stronger dimer than that established by the RpiB structure. The four residues Ile-133, Met-137, Leu-138, and Met 141 of α6 in LacA form hydrophobic interactions with Tyr-170, Trp-165, Leu-162, Phe-158, and Phe-157 of α7 and Tyr-42, Pro-43, and Ile-44 of α2 in LacB. Water molecules are not present at these interactions.

### Active site pocket between LacA and LacB

**Figure 3:**
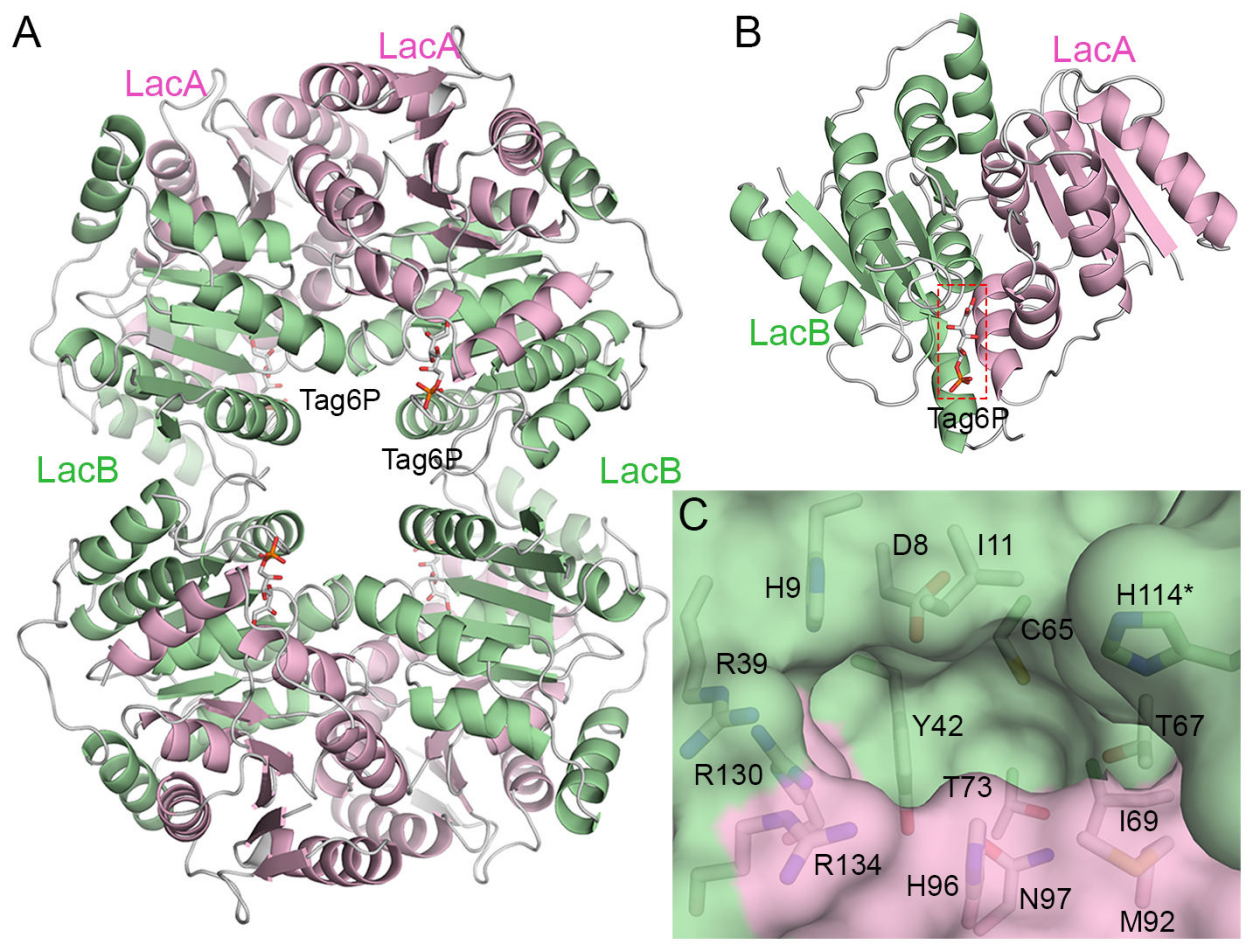
The active site pocket in LacAB. **A**, The four active sites in the whole LacAB structure are located at the inside of LacAB homotetramer. **B**, Based on the structure of the LacAB complex with the product tagatose-6-phosphate (Tag6P), the substrate-binding site of LacAB is at the interface between LacA and LacB subunits. **C**, A close-up surface representation of the active site at the interface shows a deep, wide-mouthed, trapezoid-shaped cavity formed by the residues Met-92, His-96, Asn-97, Arg-130, His-131, and Arg-134 in LacA, and Asp-8, His-9, Ile-10, Arg-39, Tyr-42, Cys-65, Thr-67, Ile-69, and Thr-73 in LacB. Residue of LacB in other neighboring LacAB is indicated by asterisks.

**Figure 4:**
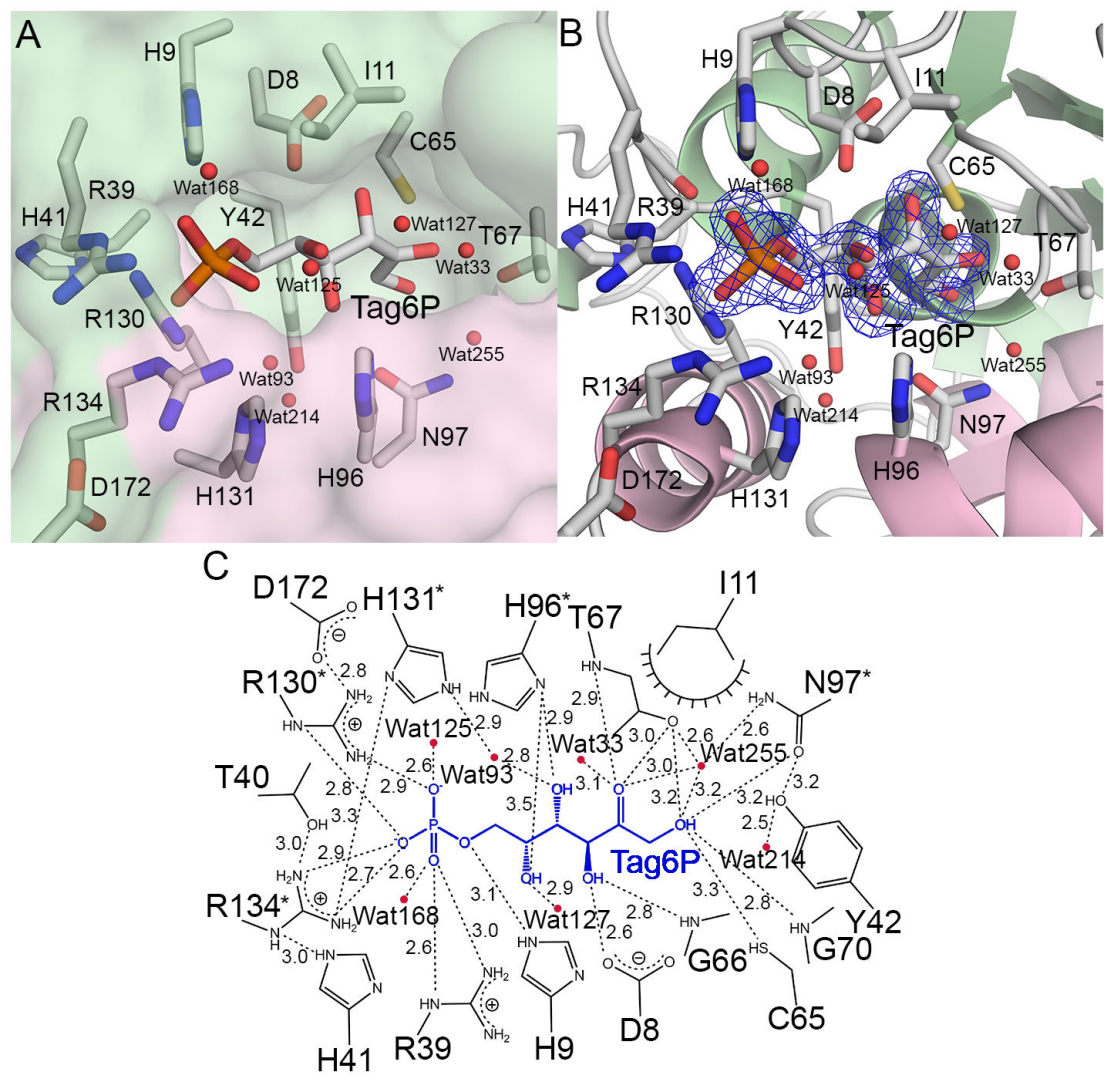
The active site in the LacAB-Tag6P complex. **A**, Surface representation of the active site showing the binding orientation of tagatose-6-phosphate in the pocket. **B**, The final 2Fo-Fc electron density map contoured at 1.0 σ and overlaid on the model for tagatose-6-phosphate and water molecules (red spheres) binding in the active site pocket of LacAB. **C**, Schematic showing the detailed binding mode of tagatose-6-phosphate (blue) in the active site. Dashed lines indicate hydrogen bondings and polar interactions, which are labeled with the interatomic distances in Å. Decorated arcs represent van der Waals interactions of less than 5.0 Å. Water molecules are shown as red circles. Residues of LacA are indicated by asterisks.

A structural comparison between LacAB and RpiB by CCP4MG analysis suggested that the active site of LacAB is at the interface of each LacA and LacB subunit [25]. To verify this, the structure of LacAB in a complex with D-tagatose-6-phosphate (Tag6P) as a product was determined in a soaking experiment (Figure 3A and B). The active site pocket was confirmed by the electron density of Tag6P in a wide-mouthed trapezoid-shaped cavity, 10 Å deep, between LacA and LacB (Figure 3C). In contrast to the two substrate-binding sites of the RpiB homodimer (Figure S3A), only one active site was verified in the LacAB monomer based on the Tag6P electron density map (Figure 4A and B). The other vacant and shallow site corresponding to the RpiB was consisted of entirely different residues from the active site, and occupied by hydrophobic residues Phe-40 (LacA), Leu-99 and Leu-137 (LacB) plus bulky residues Tyr-65 (LacA) and Tyr-94 (LacB). The active site is located at the interface surrounding two helices (α4 and α6) in LacA and the β1/α1 loop, α2, and β3/α3 loop in LacB. The tunnel in a narrow side of the dent, which was disclosed in the structure of the active site of the *Mt*RpiB homodimer, is sealed off by His-114 of α5 in LacB of another LacAB (Figure 3C). The pocket is composed of residues Met-92, His-96, Asn-97, Arg-130, His-131, and Arg-134 in LacA plus Asp-8, His-9, Ile-10, Arg-39, Tyr-42, Cys-65, Thr-67, Ile-69, and Thr-73 in LacB (Figure 3C).

The Superpose program [26] of the CCP4 suite [27] did not demonstrate any structural differences in conformation between substrate-free LacAB and its complex with Tag6P; the root mean square deviation was 0.12 Å for all Cα atoms. Figure 4C shows the details of the active site in the LacAB-Tag6P complex. The binding of Tag6P in the complex is stabilized by many hydrogen bonds with hydrophilic residues in the active site pocket. The phosphate group of Tag6P is bound by bidentate or tridentate hydrogen bonds to the guanidinium group of three arginine residues (Arg-130 and Arg-134 in LacA and Arg-39 in LacB) at average distances of 2.9, 2.8, and 3.0 Å, respectively. Additionally, the phosphate group of Tag6P, which is O6-linked to C6 of D-tagatose, forms a 3-Å polar interaction with the side chain of His-9 (LacB). The O3 hydroxyl group of Tag6P interacts with the carboxyl group of Asp-8 (LacB) and the main chain of Gly-66 (LacB), while the O4 hydroxyl group interacts with the imidazole group of His-96 (LacA). The terminal O1 hydroxyl group interacts with the side chain of Cys-65 (LacB) and the amide nitrogen of Gly-70 (LacB) at distances of 3.3 and 2.8 Å, respectively. The O2 carbonyl interacts with the amide nitrogen of Thr-67 (LacB) at 2.9 Å. The carboxamide group of Asn-97 (LacA) and the hydroxyl group of Thr-67 (LacB) are directly connected to O1 and O2 of Tag6P and simultaneously related by hydrogen bonds mediated by a water molecule.

### Substrate specificity of LacAB

**Figure 5:**
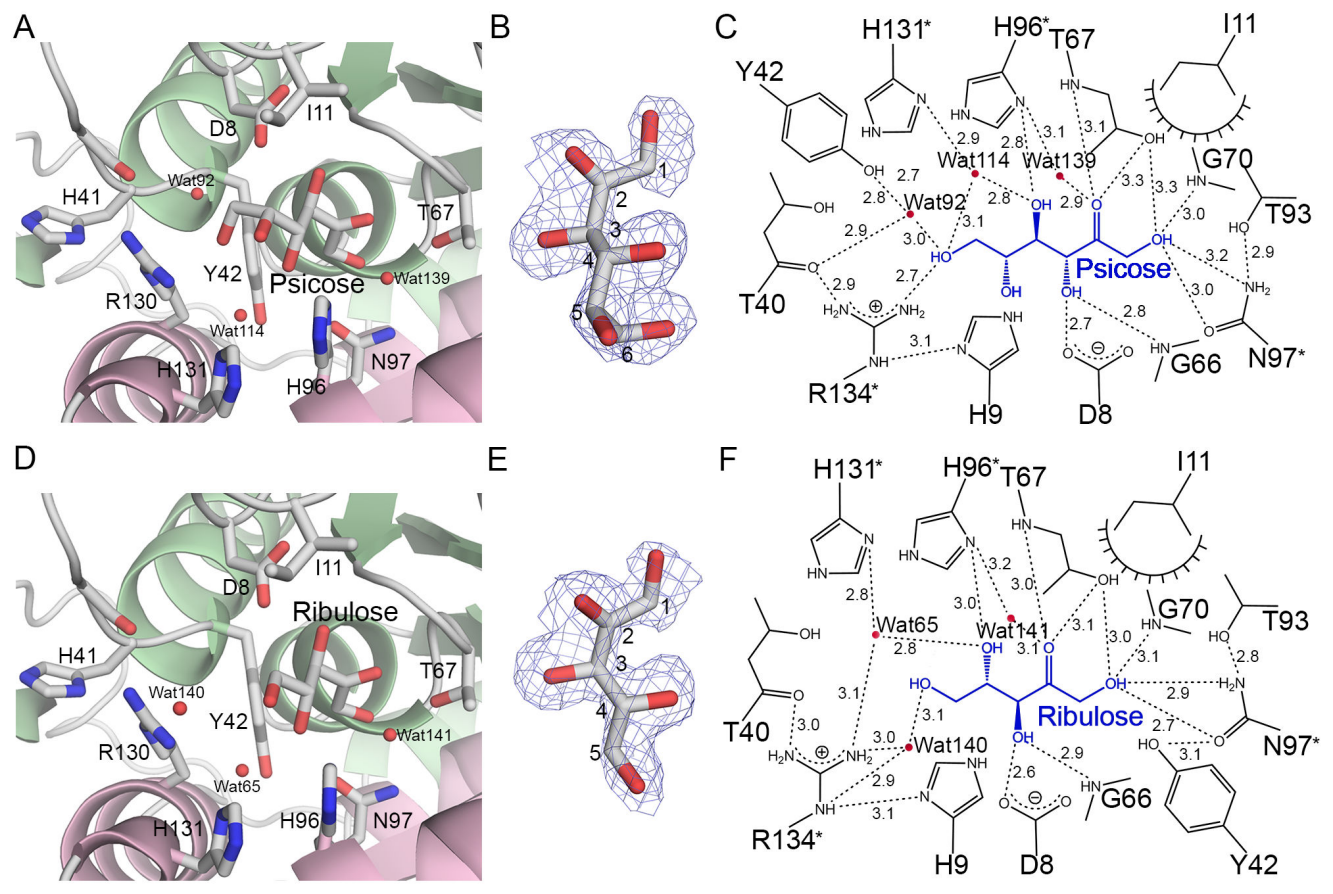
Substrate-specific binding of D-psicose and D-ribulose to LacAB. **A** and **D**, The binding of D-psicose and D-ribulose, respectively, at the active site of LacAB is shown, including the amino acid residues and water molecules (red spheres). **B** and **E**, The final 2Fo-Fc electron density maps contoured at 0.8σ are overlaid on the models for D-psicose and D-ribulose. **C** and **F**, The binding modes of D-psicose and D-ribulose. The substrates are shown in blue. Dashed lines indicate hydrogen bondings and polar interactions, which are labeled with the interatomic distances in Å. Decorated arcs represent van der Waals interactions of less than 5.0 Å. Water molecules are shown as red circles.

The structures of the LacAB complex with D-allose and D-ribose revealed that the substrates are converted to D-psicose and D-ribulose, respectively, which bind in the same orientation as Tag6P in the active site pocket (Figure 5A and D). The electron density maps of D-psicose and D-ribulose were unambiguously observed (Figure 5B and E). To identify and confirm the bound substances in LacAB complex, D-psicose and D-ribulose as the products were substituted with D-allose and D-ribose as the substrates, as well as D-allose and D-ribose as the products by ketose conversion, respectively. However, these substitutions were revealed higher B-factors for ligand after refinement. The interactions between LacAB and the bound sugars are shown schematically in Figure 5C and F. The O1, O3, and O4 hydroxyl groups as well as the O2 carbonyl group of the substrates interact with the same LacAB residues as seen in the LacAB-Tag6P complex. Additionally, the imidazole group of His-96 (LacA) directly binds to the O4 hydroxyl group of D-ribulose via hydrogen bonding at a distance of 3.0 Å. In the LacAB-psicose complex, the O6 hydroxyl group is 2.7 Å from the guanidinium group of Arg-134 (LacA).

### Functional studies using mutants of LacAB

Site-directed mutagenesis was performed to study the functional roles of LacAB residues that interact directly with Tag6P. The enzymatic activities of the mutant proteins were determined by a colorimetric method using cysteine-carbazole-sulfuric acid to measure aldose conversion from D-ribose to D-ribulose (Table S1). The catalytic activity of the T67A (LacB) mutant, which interacted with the O1 hydroxyl and O2 carbonyl of Tag6P, was approximately 20-fold lower than that of the wild-type enzyme. The H96A (LacA) mutant, which bound to the O4 and O5 hydroxyl groups by polar ineteraction at distances of 2.9 Å and 3.5 Å, respectively, exhibited 25-fold lower activity compared with the wild-type enzyme activity. The N97A (LacA) mutant bound to the terminal O1 hydroxyl group of Tag6P and had an activity level of approximately 30% that of the wild-type enzyme. Three mutants showed no enzymatic activity: the C65A (LacB) mutant, which interacted with the terminal O1 hydroxyl group; the D8N (LacB) mutant, which interacted with the O3 hydroxyl group; and the H9A (LacB) mutant, which interacted with the O6 hydroxyl group.

### Comparison between the LacAB and RpiB active sites

Structurally, LacA and LacB belong to the RpiB/LacAB superfamily and have approximately 26% sequence identity with RpiB (Figure 1B), which converts ribose-5-phosphate to ribulose-5-phosphate in the pentose phosphate pathway. Overall, the structure of LacAB is analogous to that of RpiB, except that the C-terminal α7 helix of LacB, which binds the phosphate group, is not present in RpiB and LacAB has only one active site, which is located in the same position as one of the two RpiB active sites. A comparison between the LacAB active site identified by substrate binding and one of the active sites of *Mt*RpiB from M. tuberculosis (PDB ID 2VVP) showed that the only structural difference between the two sites is the substitution of the phosphate-binding residue Arg-113 in β5/α5 of *Mt*RpiB by Arg-39 in β2/α2 of LacB (Figure S3B). Another putative pocket region of LacAB was identified by the program PocketPicker [28] and by structural similarity with the substrate-binding site of *Mt*RpiB. However, this putative pocket did not show the electron density of Tag6P and has no sequence similarity with the *Mt*RpiB site.

## Discussion

Galactose-6-phosphate isomerase (LacAB) is encoded by the tagatose-6-phosphate pathway gene cluster and belongs to the same family as RpiB. A structural alignment produced using the DALI server [29] revealed that LacAB is structurally similar to RpiB, with a Z-score higher than 20. Superimposition of the LacAB structure onto that of RpiB from E. coli (PDB ID 2VVR) gave a the root-mean-square deviation of 1.2 Å for 564 Cα atoms of a tetramer. The crystal structures of RpiB with and without substrates have been reported for various species.

Simultaneous expression of LacA and LacB proteins resulted in the formation of heterodimeric complex, and no homodimeric complex, despite structural similarities between the two proteins. The only structural difference between LacA and LacB is the additional C-terminal α-helix in LacB (Figure S3C), making LacB 30 amino acids longer than LacA and RpiB proteins. LacA and LacB expressed separately as well as their combined extracts were found to have no galactose-6-phosphate isomerase activity [5]. Therefore, we considered that the heterodimerization of LacA and LacB is more favorable than homodimeric formation because of the interaction between the C-terminal α7 helix of LacB and the α6 helix of LacA. Although LacA and LacB have similar motifs and structures, they share only 17% sequence identity, which is lower than their identity with RpiB. In particular, the active site residues differ between LacA and LacB, excluding Gly-66 and Gly-70 in LacB, which are predicted to be involved in structural conformation.

The active site of LacAB has two structural features responsible for a substrate orientation distinct from that in RpiB. The active site of LacAB is coterminous with that of RpiB, except that Arg-39 (LacB) of LacAB replaces Asp-42 of *Mt*RpiB and interacts with the phosphate group of hexose phosphate, instead of Arg-113 in *Mt*RpiB [30,31]. LacAB and RpiB also differ with respect to the shape and size of the substrate-binding pocket. At approximately 15 Å in length, the pocket in LacAB is 3 Å longer than the pocket in RpiB and is sufficient to accommodate hexose phosphate. The primary factor that determines the pocket size is the location of Arg-130 (LacA), Arg-134 (LacA), and Arg-39 (LacB) to form the outside frame of the pocket. These three long and flexible arginine side chains interact with the phosphate moiety of the substrate and can change its direction away from the catalytic residue in order to accommodate the six-carbon substrate [31]. Superimposition of the LacAB and *Mt*RpiB structures showed that Gly-129 (LacA) and Arg-130 (LacA) of the α6 helix are shifted toward the C-terminal α7 helix of LacB, with the movement of Cα atoms by 2.43 Å and 2.22 Å, respectively, for an overall Cα root-mean-square deviation of 1.2 Å. Regarding pocket shape, the substrate-binding site of LacAB is wide and open, whereas that of *Mt*RpiB is tunnel shaped and covered partially by Arg-113.

Functional studies on active-site mutants of these enzymes have indicated the identities and roles of key residues. For example, His-102 and His-99 are known to be intrinsic residues for furanose ring opening of ribose-5-phosphate in *Mt*RpiB and *Ec*RpiB, respectively [30,31]. Therefore, the binding of the conserved His-96 (LacA) to O4 and O5 of Tag6P was predicted to play a role in ring opening and isomerization of the substrate. Consistent with this proposed role of optimally orienting the substrate for catalysis, the H96A (LacA) mutant of LacAB had approximately 25-fold lower catalytic activity compared with the wild-type enzyme. Comparable mutations in *Ec*RpiB (H99N) and *Tc*RpiB (H102A) from *Trypanosoma cruzi* resulted in wild-type *K*_m_ values, but 26-fold and 10-fold lower *k*_cat_ values, respectively [31,32]. The D8A and H9A mutations of Asp-8 (LacB) and His-9 (LacB), which are conserved in the RpiB/LacAB superfamily, essentially inactivated the enzyme, suggesting that these two residues are necessary for catalytic activity. His-9 (LacB) is a conserved residue in RpiB/LacAB superfamily proteins. However, the homologous histidine residue in RpiB is not involved in binding the pentose oxygen atom that is comparable to the O6 of Tag6P. In a previous study regarding the mechanism of RpiB action, Cys-65 (LacB), which interacts with the terminal O1 hydroxyl group, was proposed to be the catalytic base to accept a proton from C2 and return a proton to C1 in the final step [30]. As an exception to all other known RpiBs, the non-conserved Glu-75 of *Mt*RpiB acts as a general base. Thr-67 (LacB) has been suggested to similarly participate in proton transfer between O1 and O2 of the enediolate intermediate [31]. That study also proposed that Asn-100 of *Ec*RpiB, consistent with Asn-97 (LacA) of LacAB, may help bind and stabilize the high-energy intermediate. In addition, Tyr-42 (LacB), which presumably helps to bind the ring-form of the substrate via stacking interactions, may have an additional role in fixing the position of Asn-97 (LacA) with a hydrogen bond [31]. Thus, LacAB uses a six-carbon sugar-phosphate as a substrate, whereas the structurally similar RpiB uses a five-carbon sugar-phosphate substrate.

Phosphoglucose isomerase (PGI, EC 5.3.1.9) and phosphomannose isomerase (PMI, EC 5.3.1.8) from *Pyrobaculum aerophilum*, which both belong to the PGI superfamily, have been analyzed structurally to elucidate their substrate interactions and mechanism of action. In contrast to the structures of RpiB/LacAB superfamily proteins, the overall structures of PGI and PMI comprise two globular domains and an arm-like C-terminal tail. The active site of these enzymes is similar to that of LacAB with regard to the histidine that is involved in ring-opening residue, but it is distinctly different from the LacAB active site in that the substrate phosphate group is held by three serines with short side chains [33,34].

In the present study, the crystal structure of LacAB was determined and was shown to be similar to that of D-ribose-5-phosphate isomerase. We adduced evidence to support the proposed functions of specific amino acid residues in the mechanism of LacAB activity based on the relative activities of site-directed mutants. Detailed kinetic experiments using mutants with various active site residue mutations may expedite the clarification of the enzyme mechanism and allow enzyme modifications for the effective production of functional rare sugars.

## Materials and Methods

### Construction of LacAB

The two genes encoding galactose-6-phosphate isomerase, LacA and LacB, were isolated from *Lactobacillus rhamnosus* by polymerase chain reaction (PCR) using specific primes. The two PCR products were subcloned into *Bam*HI and *Sal*I restriction sites of the expression vector pQE-80L (Qiagen, Hilden, Germany), which contains six N-terminal His tags. To create LacAB mutants for studies of the mechanism of action, site-directed mutagenesis of substrate-binding residues of the active site was performed using the PCR primers shown in Table S2.

### Expression, purification, and crystallization of the enzyme

The recombinant plasmids containing native and mutated *lacAB* genes were transformed into *E. coli* strain BL21 (DE3). The transformed cells were grown at 37°C in Luria-Bertani (LB) medium containing ampicillin (50 mg/L) until the optical density at 600 nm reached 0.8. Then, 0.25 mM isopropyl β-D-1-thiogalactopyranoside (IPTG) was added to induce protein expression at 30°C for 6 h. The induced cells were harvested and sonicated in lysis buffer (50 mM Tris-HCl, pH 7.5 and 200 mM NaCl). After the lysates were clarified by centrifugation, the His-tagged LacAB was purified from the crude extract by immobilized metal affinity chromatography on a HisTrap column (GE Healthcare, Waukesha, WI, USA) and elution with a buffer containing 250 mM imidazole, followed by size exclusion chromatography on a Superdex 200 prep-grade column (GE Healthcare) equilibrated with 50 mM Tris-HCl, pH 7.5 and 200 mM NaCl. The purified enzyme was concentrated to 13 mg/mL using a Vivaspin 20-mL centrifugal concentrator (Sartorius).

The initial screening for crystallization was performed at 22°C with the sitting-drop vapor diffusion method using Crystal Screen (Hampton Research) and Wizard kits (Emerald BioSystems, Bedford, MA, USA). Needle-shaped crystals were obtained from 0.2 M potassium formate containing 20% PEG 3350. High-resolution-quality and size-improved crystals were obtained from the same solution using microseeding with the hanging-drop method.

### Data collection and structure determination

Crystals of native LacAB were transferred to the crystallization solution containing 20% (v/v) glycerol as a cryoprotectant. Data of soaked native LacAB crystals were obtained at a single wavelength to 1.96 Å resolution at 100K in liquid nitrogen on beamline 7A at Pohang Accelerator Laboratory, Pohang, Korea. To obtain crystals of the LacAB-tagatose-6-phosphate complex, a LacAB crystal was soaked in crystallization solution containing 10 mM D-tagatose-6-phosphate for 30 min. Crystals of LacAB with substrates were obtained by soaking LacAB crystals in cryoprotectant solution containing 45 mM D-ribose and 40 mM D-allose for 2 hr, respectively. Single-wavelength data of the crystal complexes were collected at 1.65 Å resolution. All crystals had the space group P2 _1_2 _1_2 with unit cell parameters of a = 108.3 Å, b = 116.0 Å, c = 54.7 Å, and α = β = γ = 90°. The X-ray diffraction data were integrated and scaled with the program HKL2000 [35] (Table S3).

The crystal structures of native LacAB and the LacAB-product complex were determined by molecular replacement based on ligand-free ribose-5-phosphate isomerase (RpiB; PDB ID 1NN4) originating from *E. coli* as a search model; this protein shares 26% amino acid sequence identity with LacA and LacB according to a BLAST search, respectively. The structures of the two monomers were solved by rotation and translation searches using the program Phaser in the Phenix software suite [36], and an electron density map of the whole LacAB molecule was obtained. Owing to its uniqueness, the C-terminal 30-residue segment of LacB could not be structurally described through molecular replacement, and further model building was performed manually using the program Coot [37]. Refinement by the CNS program [38] produced the final model, with *R* and *R*_*free*_ values of 19.8% and 22.8%, respectively. Tagatose-6-phosphate in the LacAB-product complex was located based on the Fo-Fc electron density map calculated by molecular replacement using CNS. The stereochemistry of the refined structure was evaluated using ProCheck software [39]. The figures were prepared using PyMOL (PyMOL Molecular Graphics System by W.L. Delano), and the structures were analyzed using the CCP4 software suite [27]. Details of the data collection and structural refinement are shown in Table S3. The atomic coordinates and structure factors (codes 4LFK for the free form of LacAB, 4LFL for its complex with D-tagatose-6-phosphate, 4LFM for its complex with D-psicose, and 4LFN for its complex with D-ribulose) have been deposited in the Protein Data Bank (http://www.rcsb.org).

### Online size exclusion chromatography (SEC) coupled with multi-angle light scattering (MALS)

Online size exclusion chromatography (SEC) coupled with multi-angle light scattering (MALS) with embedded LS (light scattering) and UV detectors were used in the determination of molar mass in the purified LacAB. Chromatographic separation was achieved under isocratic conditions using an analytical Superdex 200 10/300 GL column connected to fast protein liquid chromatography (FPLC) with 50 mM Tris-HCl, pH 7.5 and 200 mM NaCl buffer at a flow rate of 1 mL/min. UV detector in FPLC was equipped sequentially with a MALS detector (Wyatt Technology Corporation, Santa Barbara, CA) applied a laser light at 658 nm and 18 detectors. The signals from UV and MALS were imported into Unicorn 5.11 and Astra V 5.3.4 software that was used for processing and analyzing the MALS data.

### Activity assay of LacAB and mutants

Substrate isomerization and product accumulation in the reaction mixture were determined using a colorimetric method [40]. Relative activities of LacAB and various active-site mutants were measured using 25–200 mM D-ribose as the substrate. Cysteine hydrochloride, sulfuric acid, and an alcoholic solution of carbazole were added in serial order to the reaction mixture, which contained the generated D-ribulose. After incubation at room temperature, the absorbance of the mixture was measured at the wavelength of 560 nm.

## Supporting Information

Figure S1 Oligomerization state of LacAB. The molecular characteristics of LacAB analyzed using analytical size exclusion chromatography (SEC) with online laser light scattering of multi-angle light scattering (MALS) and UV detector of fast protein liquid chromatography (FPLC). The figure shows UV280nm (blue *line*)-LS(90 angle; red *line*) overlay of LacAB homotetramer by SEC-MALS.
(TIF)

Figure S2 The interface between LacA and LacB subunits. Interacting residues between LacA and LacB subunits, shown in a side view, are located in perpendicularly stacked side chains of the respective α3/β4/α4 fold in LacA and LacB subunits. The residues are labeled.
(TIF)

Figure S3 Structural comparison between LacAB and *Mt*RpiB (PDB ID 2VVP). **A**, Two substrate-binding sites containing D-ribose-5-phosphate are present in the *Mt*RpiB honodimer. **B**, Superimposition of the LacAB and *Mt*RpiB structures shows the structural differences between their active sites. The given in brackets indicate the RpiB residues that are different between RpiB and LacAB. **C**, Differences among the structures of LacA (pink), LacB (green), and *Mt*RpiB (yellow): LacB contains an extra helix, α7, at its C-terminus, and the β2/α2 loop of LacA is oriented toward the inside of the molecule. This figure was prepared using the Superpose program [25].
(TIF)

Table S1 Relative activity of LacAB mutants. (DOCX)

Table S2 Primer sequences for mutants. (DOCX)

Table S3 Crystallographic data and refinement statistics. (DOCX)

## References

[1] Bissett DL, Anderson RL (1974) Lactose and _D_-galactose metabolism in group N streptococci: presence of enzymes for both the _D_-galactose 1-phosphate and D-tagatose 6-phosphate pathways. J Bacteriol 117: 318–320. PubMed: 4358045. 4358045 10.1128/jb.117.1.318-320.1974 PMC246560

[2] Morse ML, Hill KL, Egan JB, Hengstenberg W (1968) Metabolism of lactose by Staphylococcus aureus and its genetic basis. J Bacteriol 95: 2270–2274. PubMed: 5669899. 5669899 10.1128/jb.95.6.2270-2274.1968 PMC315162

[3] Bissett DL, Anderson RL (1974) Genetic evidence for the physiological significance of the _D_-tagatose 6-phosphate pathway of lactose and _D_-galactose degradation in staphylococcus aureus. J Bacteriol 119: 698–704. PubMed: 4277494. 4277494 10.1128/jb.119.3.698-704.1974 PMC245671

[4] Bissett DL, Wenger WC, Anderson RL (1980) Lactose and _D_-galactose metabolism in Staphylococcus aureus. II. Isomerization of _D_-galactose 6-phosphate to _D_-tagatose 6-phosphate by a specific _D_-galactose-6-phosphate isomerase. J Biol Chem 255: 8740–8744. PubMed: 7410391 7410391

[5] van Rooijen RJ, van Schalkwijk S, de Vos WM (1991) Molecular cloning, characterization, and nucleotide sequence of the tagatose 6-phosphate pathway gene cluster of the lactose operon of Lactococcus lactis. J Biol Chem 266: 7176–7181. PubMed: 1901863. 1901863

[6] de Vos WM, Vaughan EE (1994) Genetics of lactose utilization in lactic acid bacteria. FEMS Microbiol Rev 15: 217–237. doi:10.1016/0168-6445(94)90114-7. PubMed: 7946468. 7946468 10.1111/j.1574-6976.1994.tb00136.x

[7] Fridovich-Keil JL (2006) Galactosemia: the good, the bad, and the unknown. J Cell Physiol 209: 701–705. doi:10.1002/jcp.20820. PubMed: 17001680. 17001680 10.1002/jcp.20820

[8] Frey PA (1996) The Leloir pathway: a mechanistic imperative for three enzymes to change the stereochemical configuration of a single carbon in galactose. FASEB J 10: 461–470. PubMed: 8647345. 8647345

[9] Rosey EL, Oskouian B, Stewart GC (1991) Lactose metabolism by Staphylococcus aureus: characterization of *lacABCD*, the structural genes of the tagatose 6-phosphate pathway. J Bacteriol 173: 5992–5998. PubMed: 1655695. 1655695 10.1128/jb.173.19.5992-5998.1991 PMC208343

[10] Rosey EL, Stewart GC (1992) Nucleotide and deduced amino acid sequences of the *lacR*, *lacABCD*, and *lacFE* genes encoding the repressor, tagatose 6-phosphate gene cluster, and sugar-specific phosphotransferase system components of the lactose operon of Streptococcus mutans. J Bacteriol 174: 6159–6170. PubMed: 1400164. 1400164 10.1128/jb.174.19.6159-6170.1992 PMC207683

[11] Jagusztyn-Krynicka EK, Hansen JB, Crow VL, Thomas TD, Honeyman AL et al. (1992) Streptococcus mutans serotype c tagatose 6-phosphate pathway gene cluster. J Bacteriol 174: 6152–6158. PubMed: 1328153. 1328153 10.1128/jb.174.19.6152-6158.1992 PMC207682

[12] Zeng L, Martino NC, Burne RA (2012) Two gene clusters coordinate galactose and lactose metabolism in Streptococcus gordonii. Appl Environ Microbiol 78: 5597–5605. doi:10.1128/AEM.01393-12. PubMed: 22660715. 22660715 10.1128/AEM.01393-12 PMC3406145

[13] Anderson RL, Bissett DL (1982) _D_-Galactose-6-phosphate isomerase. Methods Enzymol 89 Pt D: 562–565 PubMed: 7144590. 10.1016/s0076-6879(82)89097-6 7144590

[14] Miallau L, Hunter WN, McSweeney SM, Leonard GA (2007) Structures of Staphylococcus aureus _D_-tagatose-6-phosphate kinase implicate domain motions in specificity and mechanism. J Biol Chem 282: 19948–19957. doi:10.1074/jbc.M701480200. PubMed: 17459874. 17459874 10.1074/jbc.M701480200

[15] Hall DR, Bond CS, Leonard GA, Watt CI, Berry A et al. (2002) Structure of tagatose-1,6-bisphosphate aldolase. Insight into chiral discrimination, mechanism, and specificity of class II aldolases. J Biol Chem 277: 22018–22024. 11940603 10.1074/jbc.M202464200

[16] LowKam C, Liotard B, Sygusch J (2010) Structure of a class I tagatose-1,6-bisphosphate aldolase: investigation into an apparent loss of stereospecificity. J Biol Chem 285: 21143–21152. doi:10.1074/jbc.M109.080358. PubMed: 20427286. 20427286 10.1074/jbc.M109.080358 PMC2898290

[17] Zhang R-G, Andersson CE, Skarina T, Evdokimova E, Edwards AM et al. (2003) The 2.2 A resolution structure of RpiB/AlsB from Escherichia coli illustrates a new approach to the ribose-5-phosphate isomerase reaction. J Mol Biol 332: 1083–1094. doi:10.1016/j.jmb.2003.08.009. PubMed: 14499611. 14499611 10.1016/j.jmb.2003.08.009 PMC2792017

[18] Park C-S, Yeom S-J, Kim HJ, Lee S-H, Lee J-K et al. (2007) Characterization of ribose-5-phosphate isomerase of Clostridium thermocellum producing _D_-allose from _D_-psicose. Biotechnol Lett 29: 1387–1391. doi:10.1007/s10529-007-9393-7. PubMed: 17484020. 17484020 10.1007/s10529-007-9393-7

[19] Yoon R-Y, Yeom S-J, Kim HJ, Oh D-K (2009) Novel substrates of a ribose-5-phosphate isomerase from Clostridium thermocellum. J Biotechnol 139: 26–32. doi:10.1016/j.jbiotec.2008.09.012. PubMed: 18984017. 18984017 10.1016/j.jbiotec.2008.09.012

[20] Park H-Y, Park C-S, Kim HJ, Oh D-K (2007) Substrate specificity of a galactose 6-phosphate isomerase from Lactococcus lactis that produces d-allose from d-psicose. J Biotechnol 132: 88–95. doi:10.1016/j.jbiotec.2007.08.022. PubMed: 17868944. 17868944 10.1016/j.jbiotec.2007.08.022

[21] Yeom S-J, Ji J-H, Kim N-H, Park C-S, Oh D-K (2009) Substrate specificity of a mannose-6-phosphate isomerase from Bacillus subtilis and its application in the production of L -ribose. Appl Environ Microbiol 75: 4705–4710. doi:10.1128/AEM.00310-09. PubMed: 19447949. 19447949 10.1128/AEM.00310-09 PMC2708437

[22] Krissinel E, Henrick K (2007) Inference of macromolecular assemblies from crystalline state. J Mol Biol 372: 774–797. doi:10.1016/j.jmb.2007.05.022. PubMed: 17681537. 17681537 10.1016/j.jmb.2007.05.022

[23] Rangarajan ES, Sivaraman J, Matte A, Cygler M (2002) Crystal structure of _D_-ribose-5-phosphate isomerase (RpiA) from Escherichia coli. Proteins 48: 737–740. doi:10.1002/prot.10203. PubMed: 12211039. 12211039 10.1002/prot.10203

[24] Zhang R-G, Andersson CE, Savchenko A, Skarina T, Evdokimova E et al. (2003) Structure of Escherichia coli ribose-5-phosphate isomerase: a ubiquitous enzyme of the pentose phosphate pathway and the Calvin cycle. Structure 11: 31–42. doi:10.1016/S0969-2126(02)00933-4. PubMed: 12517338. 12517338 10.1016/s0969-2126(02)00933-4 PMC2792023

[25] McNicholas S, Potterton E, Wilson KS, Noble MEM (2011) Presenting your structures: the CCP4mg molecular-graphics software. Acta Crystallogr D Biol Crystallogr 67: 386–394. doi:10.1107/S0907444911007281. PubMed: 21460457. 21460457 10.1107/S0907444911007281 PMC3069754

[26] Krissinel E, Henrick K (2004) Secondary-structure matching (SSM), a new tool for fast protein structure alignment in three dimensions. Acta Crystallogr D Biol Crystallogr 60: 2256–2268. doi:10.1107/S0907444904026460. PubMed: 15572779. 15572779 10.1107/S0907444904026460

[27] Winn MD, Ballard CC, Cowtan KD, Dodson EJ, Emsley P et al. (2011) Overview of the CCP4 suite and current developments. Acta Crystallogr D Biol Crystallogr 67: 235–242. doi:10.1107/S0907444910045749. PubMed: 21460441. 21460441 10.1107/S0907444910045749 PMC3069738

[28] Weisel M, Proschak E, Schneider G (2007) PocketPicker: analysis of ligand binding-sites with shape descriptors. Chem Cent J 1: 7. doi:10.1186/1752-153X-1-7. PubMed: 17880740. 17880740 10.1186/1752-153X-1-7 PMC1994066

[29] Holm L, Rosenström P (2010) Dali server: conservation mapping in 3D. Nucleic Acids Res 38: W545–W549. doi:10.1093/nar/gkp893. PubMed: 20457744. 20457744 10.1093/nar/gkq366 PMC2896194

[30] Roos AK, Andersson CE, Bergfors T, Jacobsson M, Karlén A et al. (2004) Mycobacterium tuberculosis ribose-5-phosphate isomerase has a known fold, but a novel active site. J Mol Biol 335: 799–809. doi:10.1016/j.jmb.2003.11.021. PubMed: 14687575. 14687575 10.1016/j.jmb.2003.11.021

[31] Roos AK, Mariano S, Kowalinski E, Salmon L, Mowbray SL (2008) _D_-ribose-5-phosphate isomerase B from Escherichia coli is also a functional _D_-allose-6-phosphate isomerase, while the Mycobacterium tuberculosis enzyme is not. J Mol Biol 382: 667–679. doi:10.1016/j.jmb.2008.06.090. PubMed: 18640127. 18640127 10.1016/j.jmb.2008.06.090

[32] Stern AL, Burgos E, Salmon L, Cazzulo JJ (2007) Ribose 5-phosphate isomerase type B from Trypanosoma cruzi: kinetic properties and site-directed mutagenesis reveal information about the reaction mechanism. Biochem J 401: 279–285. doi:10.1042/BJ20061049. PubMed: 16981853. 16981853 10.1042/BJ20061049 PMC1698680

[33] Swan MK, Hansen T, Schönheit P, Davies C (2004) A novel phosphoglucose isomerase (PGI)/phosphomannose isomerase from the crenarchaeon Pyrobaculum aerophilum is a member of the PGI superfamily: structural evidence at 1.16-A resolution. J Biol Chem 279: 39838–39845. doi:10.1074/jbc.M406855200. PubMed: 15252053. 15252053 10.1074/jbc.M406855200

[34] Swan MK, Hansen T, Schönheit P, Davies C (2004) Structural basis for phosphomannose isomerase activity in phosphoglucose isomerase from Pyrobaculum aerophilum: a subtle difference between distantly related enzymes. Biochemistry 43: 14088–14095. doi:10.1021/bi048608y. PubMed: 15518558. 15518558 10.1021/bi048608y

[35] Otwinowski Z, Minor W (1997) Processing of X-ray Diffraction Data Collected in Oscillation Mode. Methods Enzymol. 276: 307-326. doi:10.1016/S0076-6879(97)76066-X. 10.1016/S0076-6879(97)76066-X 27754618

[36] Adams PD, Afonine PV, Bunkóczi G, Chen VB, Davis IW et al. (2010) PHENIX: a comprehensive Python-based system for macromolecular structure solution. Acta Crystallogr D Biol Crystallogr 66: 213–221. doi:10.1107/S0907444909052925. PubMed: 20124702. 20124702 10.1107/S0907444909052925 PMC2815670

[37] Emsley P, Cowtan K (2004) Coot: model-building tools for molecular graphics. Acta Crystallogr D Biol Crystallogr 60: 2126–2132. doi:10.1107/S0907444904019158. PubMed: 15572765. 15572765 10.1107/S0907444904019158

[38] Brünger AT, Adams PD, Clore GM, DeLano WL, Gros P et al. (1998) Crystallography & NMR system: A new software suite for macromolecular structure determination. Acta Crystallogr D Biol Crystallogr 54: 905–921. doi:10.1107/S0108767398011465. PubMed: 9757107. 9757107 10.1107/s0907444998003254

[39] Dodson EJ, Winn M, Ralph A (1997) Collaborative Computational Project, number 4: providing programs for protein crystallography. Methods Enzymol 277: 620–633. doi:10.1016/S0076-6879(97)77034-4. PubMed: 18488327. 18488327 10.1016/s0076-6879(97)77034-4

[40] Dische Z, Borenfreund E (1951) A new spectrophotometric method for the detection and determination of keto sugars and trioses. J Biol Chem 192: 583-587. PubMed: 14907652. 14907652

[41] Gouet P, Courcelle E, Stuart DI, Métoz F (1999) ESPript : Analysis of multiple sequence alignment in PostScript. Bioinformatics 15: 305–308. doi:10.1093/bioinformatics/15.4.305. PubMed: 10320398. 10320398 10.1093/bioinformatics/15.4.305

