# Supplementary figures and images for: Crystal Structure and Substrate Specificity of D-Galactose-6-Phosphate Isomerase Complexed with Substrates
Source: PLoS One. 2013 Aug 28;8(8):e72902. doi: 10.1371/journal.pone.0072902 (PMC3755991; doi:10.1371/journal.pone.0072902)

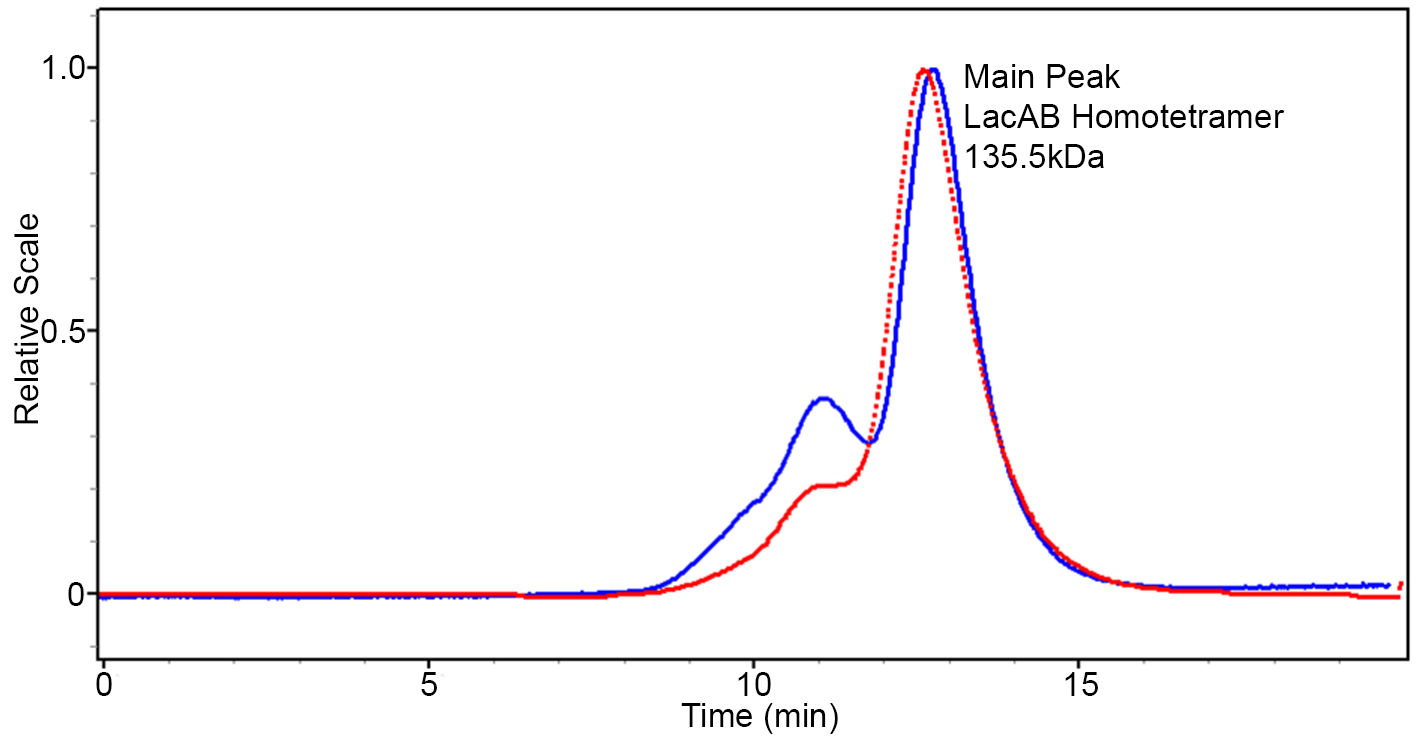

Supplement: Figure S1 — The molecular characteristics of LacAB analyzed using analytical size exclusion chromatography (SEC) with online laser light scattering of multi-angle light scattering (MALS) and UV detector of fast protein liquid chromatography (FPLC). The figure shows UV280nm (blue line)-LS(90 angle; red line) overlay of LacAB homotetramer by SEC-MALS. (TIF) [file pone.0072902.s001.tif]

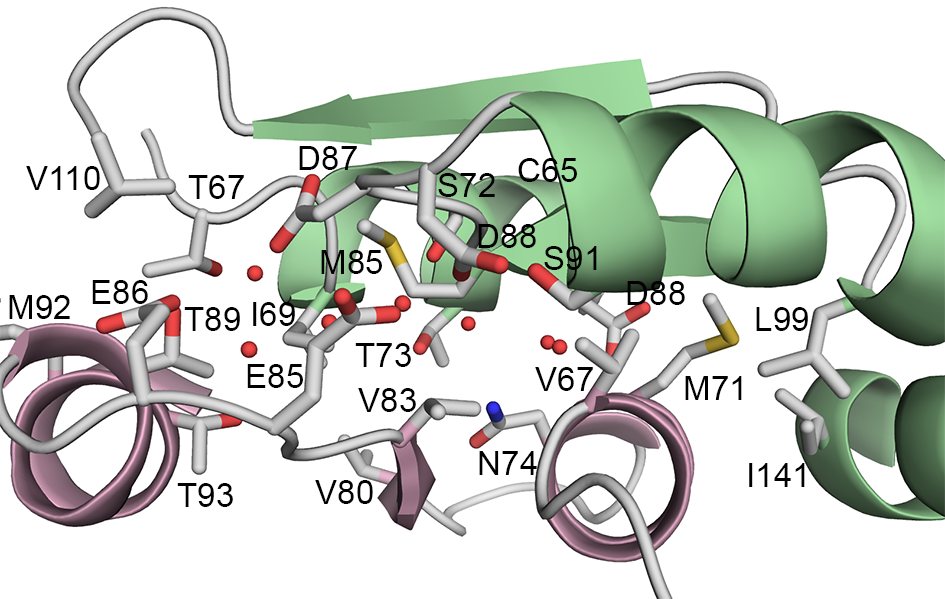

Supplement: Figure S2 — Interacting residues between LacA and LacB subunits, shown in a side view, are located in perpendicularly stacked side chains of the respective α3/β4/α4 fold in LacA and LacB subunits. The residues are labeled. (TIF) [file pone.0072902.s002.tif]

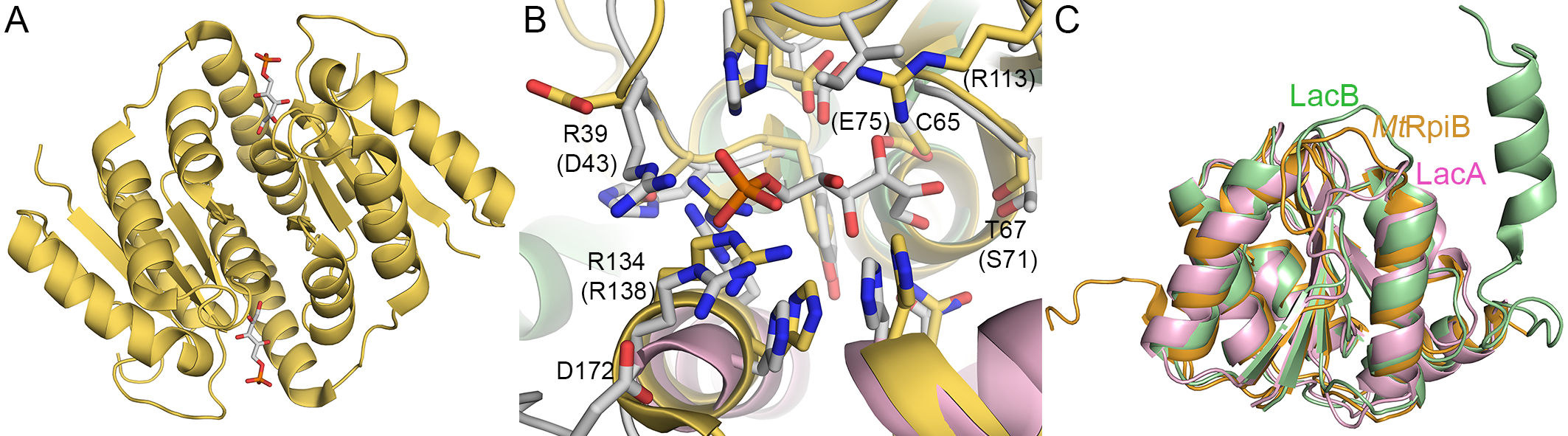

Supplement: Figure S3 — A, Two substrate-binding sites containing D-ribose-5-phosphate are present in the MtRpiB honodimer. B, Superimposition of the LacAB and MtRpiB structures shows the structural differences between their active sites. The given in brackets indicate the RpiB residues that are different between RpiB and LacAB. C, Differences among the structures of LacA (pink), LacB (green), and MtRpiB (yellow): LacB contains an extra helix, α7, at its C-terminus, and the β2/α2 loop of LacA is oriented toward the inside of the molecule. This figure was prepared using the Superpose program [25]. (TIF) [file pone.0072902.s003.tif]
