# Supplementary material for: Crystal Structure and Substrate Specificity of D-Galactose-6-Phosphate Isomerase Complexed with Substrates
Source: PLoS One. 2013 Aug 28;8(8):e72902. doi: 10.1371/journal.pone.0072902 (PMC3755991; doi:10.1371/journal.pone.0072902)
Supplement: Table S1 — (DOCX) [file pone.0072902.s004.docx]

**Table S1.** Relative activity of LacAB mutants

| **Enzyme** | **Relative activity** |
| --- | --- |
| Wild type | **+++** |
| D8N (LacB) | **-** |
| H9A (LacB) | **-** |
| C65A (LacB) | **-** |
| T67A (LacB) | **+** |
| H96A (LacA) | **+** |
| N97A (LacA) | **+** |
