# Supplementary material for: Crystal Structure and Substrate Specificity of D-Galactose-6-Phosphate Isomerase Complexed with Substrates
Source: PLoS One. 2013 Aug 28;8(8):e72902. doi: 10.1371/journal.pone.0072902 (PMC3755991; doi:10.1371/journal.pone.0072902)
Supplement: Table S2 — (DOCX) [file pone.0072902.s005.docx]

**Table S2.** Primer sequences for mutants

| **Mutants** | | **Primer** |
| --- | --- | --- |
| D8N | Forward  Reverse | 5’-CTTATGATTATTGCTATTGGTAACAATCACATCGTCACGATGC-3’  5’-GCATCGTGACGATGTGATTGTTACCAATAGCAATAATCATAAG-3’ |
| H9A | Forward  Reverse | 5’-CTTATGATTATTGCTATTGGTAACGATGCCATCGTCACGATGC-3’  5’-GCATCGTGACGATGGCATCGTTACCAATAGCAATAATCATAAG-3’ |
| C65A | Forward  Reverse | 5’-GGTATCGTGATGTGCGGTGCAGGTATCGGTATTTCAACAGC-3’  5’-GCTGTTGAAATACCGATACCTGCACCGCACATCACGATACC-3’ |
| T67A | Forward  Reverse | 5’-GGTATCGTGATGGCCGGTACAGGTATCGGTATTTCAACAGC-3’  5’-GCTGTTGAAATACCGATACCTGTACCGGCCATCACGATACC-3’ |
| H96A | Forward  Reverse | 5’-CACATGACGGCTGAAGCTAATGGCGCCAAGGCAATTGC-3’  5’-GCAATTGCCTTGGCGCCATTAGCTTCAGCCGTCATGTG-3’ |
| N97A | Forward  Reverse | 5’-CACATGACGGCTGAACATGCTGGCGCCAAGGCAATTGC-3’  5’-GCAATTGCCTTGGCGCCAGCATGTTCAGCCGTCATGTG-3’ |
