# Supplementary material for: Crystal Structure and Substrate Specificity of D-Galactose-6-Phosphate Isomerase Complexed with Substrates
Source: PLoS One. 2013 Aug 28;8(8):e72902. doi: 10.1371/journal.pone.0072902 (PMC3755991; doi:10.1371/journal.pone.0072902)
Supplement: Table S3 — (DOCX) [file pone.0072902.s006.docx]

**Table S3.** Crystallographic data and refinement statistics

|  | **apo LacAB** | **LacAB-tag6p** | **LacAB-psicose** | **LacAB-ribulose** |
| --- | --- | --- | --- | --- |
| **Data collection** |  |  |  |  |
| Spacegroup | P2_1_2_1_2 | P2_1_2_1_2 | P2_1_2_1_2 | P2_1_2_1_2 |
| Unit cell  a, b, c (Å)  α, β, γ (°) | a = 108.174  b = 115.926  c = 54.858  α = β = γ = 90 | a = 108.076  b = 116.114  c = 54.492  α = β = γ = 90 | a = 108.255  b = 115.958  c = 54.729  α = β = γ = 90 | a = 108.261  b = 116.009  c = 54.752  α = β = γ = 90 |
| Wavelength (Å) | 0.97932 | 0.97932 | 0.97932 | 0.97932 |
| Resolution (Å) | 50.0-1.96 (1.99-1.96) | 50.0-1.65 (1.68-1.65) | 50.0-1.65 (1.68-1.65) | 50.0-1.65 (1.68-1.65) |
| R_merge_^a^ (%) | 7.3 (31.9) | 6.3 (26.9) | 9.6 (56.1) | 10.2 (59.7) |
| I/σI | 43.3 (7.7) | 41.4 (9.2) | 31.6 (3.7) | 27.6 (3.6) |
| Completeness (%) | 99.7 (94.1) | 98.1 (99.7) | 98.5 (96.8) | 97.9 (95.9) |
| Redundancy | 14.5 (11.7) | 14.1 (12.2) | 12.9 (10.7) | 12.3 (10.3) |
| **Refinement** |  |  |  |  |
| Resolution (Å) | 50.0-1.96 | 50.0-1.65 | 50.0-1.65 | 50.0-1.65 |
| No. reflections (test) | 47,906 (4,856) | 78,655 (7,879) | 76,510 (7,643) | 76,172 (7,656) |
| R_work_^b^/R_free_^c^ (%) | 20.7 / 23.2 | 21.7 / 23.8 | 22.2 / 24.3 | 22.3 / 24.3 |
| No. atoms |  |  |  |  |
| Protein | 4,842 | 4,842 | 4,842 | 4,842 |
| Ligand |  | 32 | 24 | 20 |
| Water | 176 | 297 | 199 | 219 |
| Avg. B-factor (Å^2^) |  |  |  |  |
| Protein | 22.6 | 20.6 | 24.3 | 25.5 |
| Ligand |  | 23.2 | 44.1 | 45.2 |
| Water | 23.5 | 26.3 | 26.8 | 28.8 |
| RMS deviations |  |  |  |  |
| Bond lengths (Å) | 0.048 | 0.046 | 0.048 | 0.049 |
| Bond angle (º) | 1.159 | 1.184 | 1.184 | 1.207 |
| Ramachandran plot |  |  |  |  |
| Most favored (%) | 91.7 | 91.7 | 92.1 | 90.6 |
| Allowed (%) | 8.3 | 8.3 | 7.9 | 9.4 |
| Disallowed | 0 | 0 | 0 | 0 |
| PDB ID | 4LFK | 4LFL | 4LFM | 4LFN |

Values in parenthesis are for the highest resolution shell.

^a^*R*_merge_ = Σ|*I*_h_ - 〈*I*_h_〉 |/Σ *I*_h_, where *I*_h_ is the observed intensity and 〈*I*_h_〉 is the average intensity.

^b^*R*_work_ = Σ||*F*_obs_| - k|*F*_cal_||/Σ|*F*_obs_|.

^c^*R*_free_ is the same as *R*_obs_ for a selected subset (10%) of the reflections that was excluded from refinement.
